# Retraction

**DOI:** 10.3390/ijms13021804

**Published:** 2012-02-29

Zhong Ye; Darya O. Mishchuk; Natasha S. Stephens and Carolyn M. Slupsky. Dextran Sulfate Sodium Inhibits Alanine Synthesis in Caco-2 Cells. *Int. J. Mol. Sci.* 2011, *12*, 2325–2335.

**Shu-Kun Lin**

MDPI AG, Postfach, CH-4005 Basel, Switzerland; E-Mail: lin@mdpi.com

Received: 8 January 2012 / Published: 9 February 2012

It has been brought to our attention by the corresponding author that the results presented this article [1] are in error due to the fact that the media supplement glutaMAX was used in place of L-glutamine for culture of the control cells, while L-glutamine was used for culture of the treated cells. All authors have confirmed that the reported result could not be reproduced using the correct culture conditions. We would like to thank the authors for pointing out this error thereby upholding the ethics of scientific publication. The Editorial Team and Publisher have agreed with the authors that this manuscript should be retracted. We apologize for any inconvenience this may have caused.
